# Supplementary material for: Distinct transcriptomes and autocrine cytokines underpin maturation and survival of antibody-secreting cells in systemic lupus erythematosus
Source: Nat Commun. 2024 Mar 1;15:1899. doi: 10.1038/s41467-024-46053-w (PMC10907730; doi:10.1038/s41467-024-46053-w)
Supplement: Supplementary file 1 — Supplementary Information [file 41467_2024_46053_MOESM1_ESM.pdf]

Distinct transcriptomes and autocrine cytokines underpin maturation and survival of antibody-secreting cells in systemic lupus erythematosus

Authors: Weirong Chen[1], So-Hee Hong[1], Scott A. Jenks[1], Fabliha A. Anam[1], Christopher M. Tipton[1], Matthew C. Woodruff[1], Jennifer R. Hom[1], Kevin S. Cashman[1], Caterina Elisa Faliti[1], Xiaoqian Wang[1], Shuya Kyu[2], Chungwen Wei[1], Christopher D. Scharer[3], Tian Mi[3], Sakeenah Hicks[3], Louise Hartson[1], Doan C. Nguyen[2], Arezou Khosroshahi [1], Saeyun Lee[1], Youliang Wang[1], Regina Bugrovsky[1], Yusho Ishii[1], F. Eun-Hyung Lee[2], Ignacio Sanz[1].

[1] Department of Medicine, Division of Rheumatology, Lowance Center for Human Immunology, School of Medicine, Emory University, Atlanta, GA, USA
[2] Department of Medicine, Division of Pulmonary, Allergy, Critical Care and Sleep Medicine, School of Medicine, Emory University, Atlanta, GA, USA
[3] Department of Microbiology and Immunology, School of Medicine, Emory University, Atlanta, GA, USA

Table of Contents

1. Supplementary Figures
   Supplementary Figure 1: Circulating ASC and populations are correlated with SLE diseases activity index.
   Supplementary Figure 2: Blimp-1 and BCMA staining for circulating B cells and ASC populations from active SLE patients.
   Supplementary Figure 3: Immune phenotype of circulating ASC in active SLE.
   Supplementary Figure 4: IgG-expressing ASC are enriched in active SLE patients.
   Supplementary Figure 5: Gating strategy for ASC populations.
   Supplementary Figure 6: Heterogeneous SLE ASC responses share common precursors.
   Supplementary Figure 7: Clonal connectivity and somatic hypermutation analysis of SLE ASC.
   Supplementary Figure 8: SLE ASC express abnormal pathways.

2. Supplementary Table
   Supplementary Table 1: Summary statistics of repertoire analysis for ASC from active SLE samples.

a

b

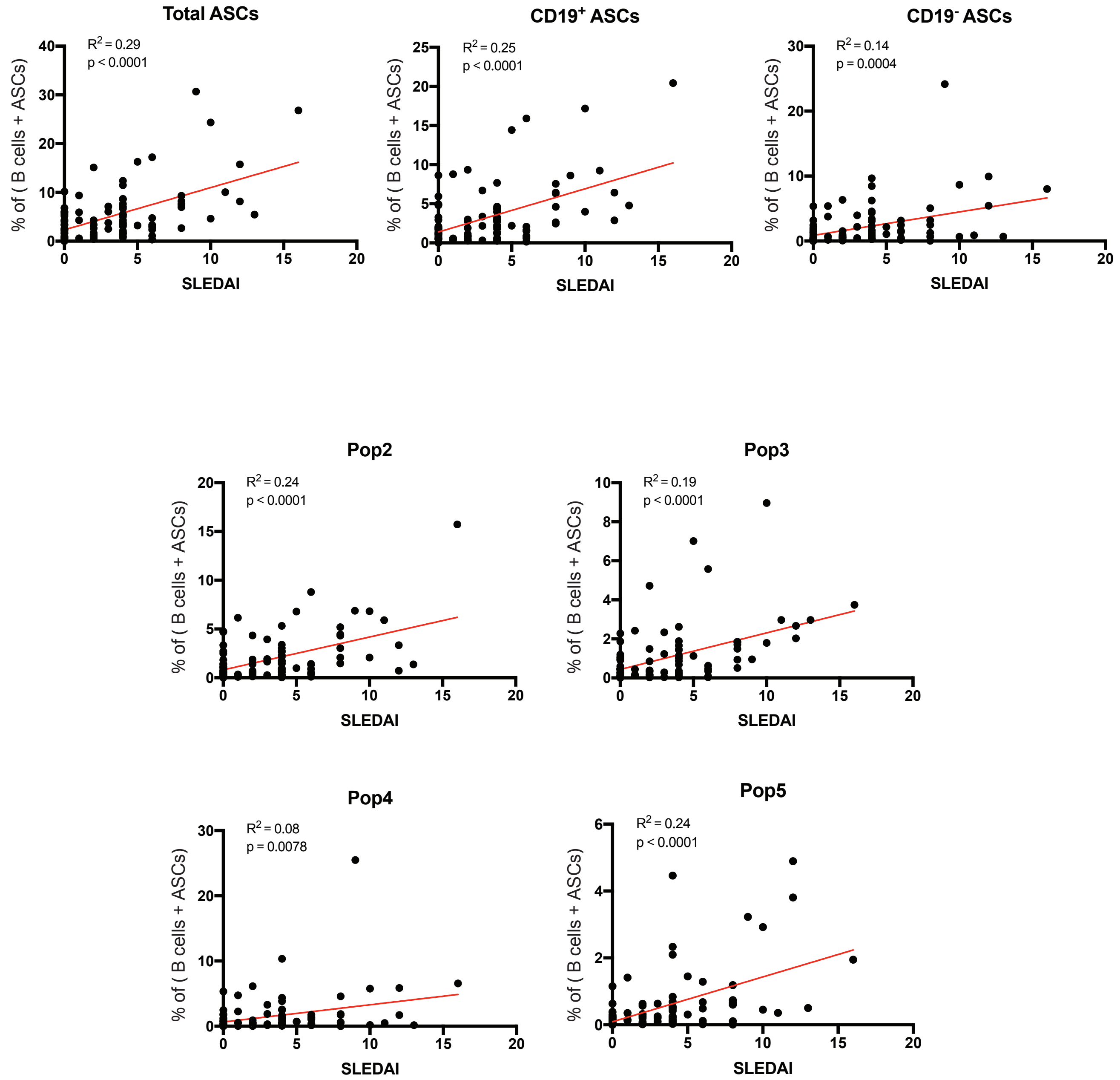

Supplementary Fig. 1: Circulating ASC and populations are correlated with SLE diseases Activity Index (SLEDAI). **a**, Positive correlation between the disease activity and frequencies of total peripheral blood ASC, CD19+ ASC, and CD19- ASC in total B cells and ASC combined using Spearman's r coefficient (*n* = 84). **b**, Positive correlation of disease activity and the frequencies of each ASC population in the peripheral blood in total B cells and ASC combined using Spearman's r coefficient (n= 84). Source data are provided as a Source Data file.

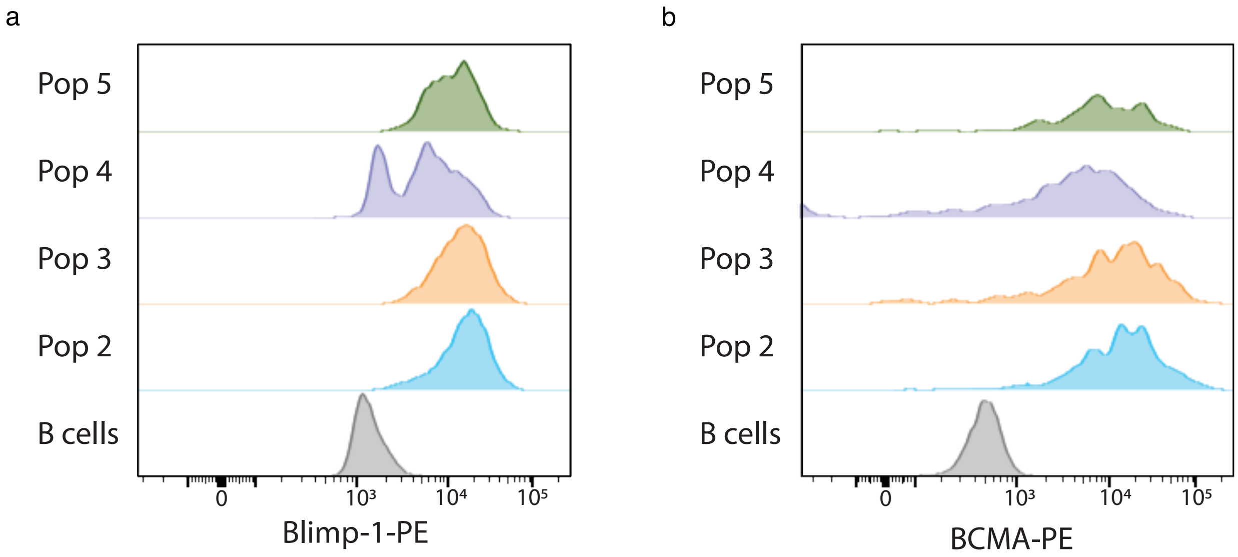

Supplementary Fig. 2: Blimp-1 and BCMA staining for circulating B cells and ASC populations from active SLE patients. **a**, Representative staining of Blimp-1 on circulating B cells and ASC populations from active SLE patients. **b**, Representative staining of BCMA on circulating B cells and ASC populations from active SLE patients.

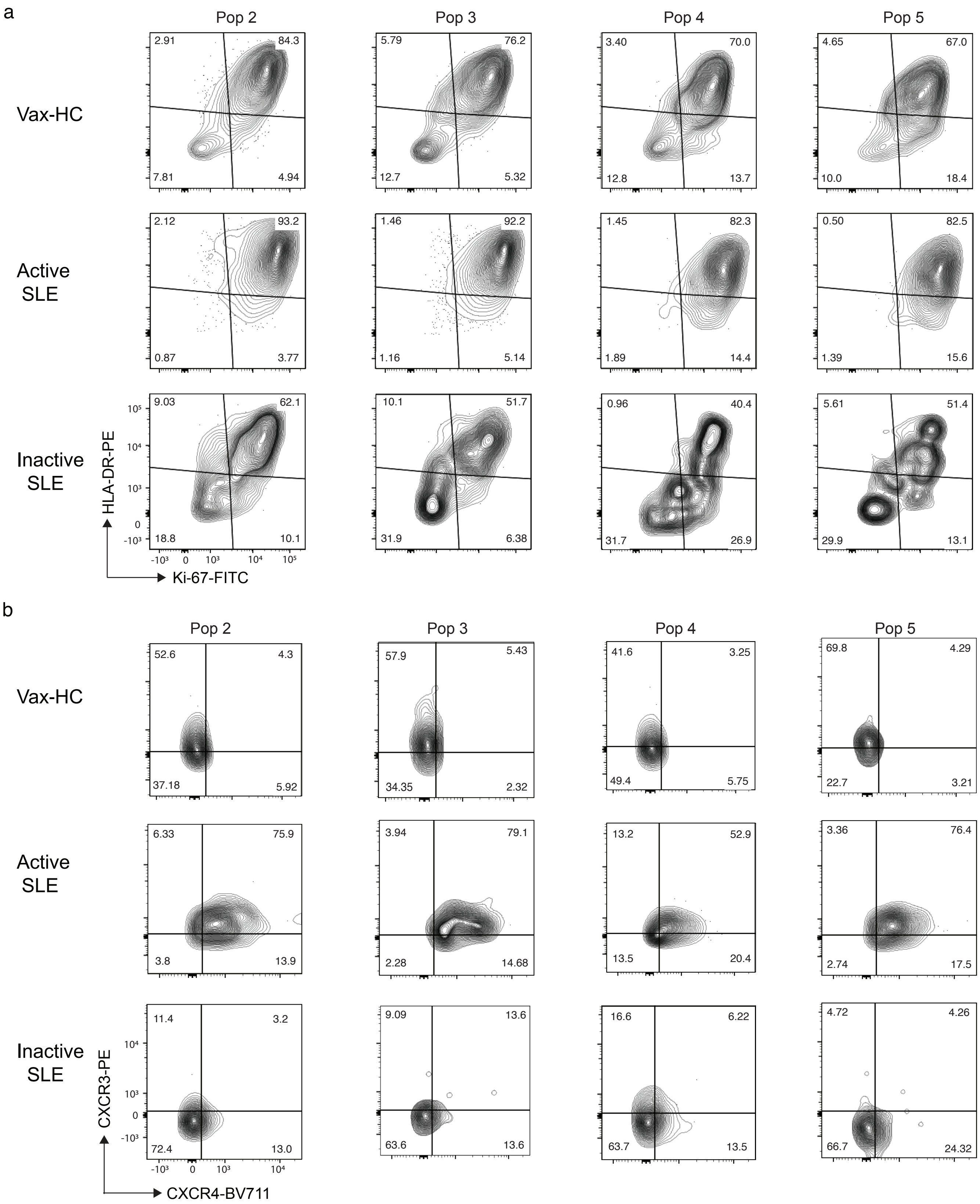

Supplementary Fig. 3: Immune phenotype of circulating ASC in active SLE. **a**, Representative co-staining of intracellular Ki-67 and surface HLA-DR on ASC populations from influenza vaccinated heathy subjects on day 7 post immunization, active SLE patients and inactive SLE patients. **b**, Representative co-staining of surface CXCR3 and CXCR4 on ASC populations from influenza vaccinated heathy subjects on day 7 post immunization, active SLE patients and inactive SLE patients.

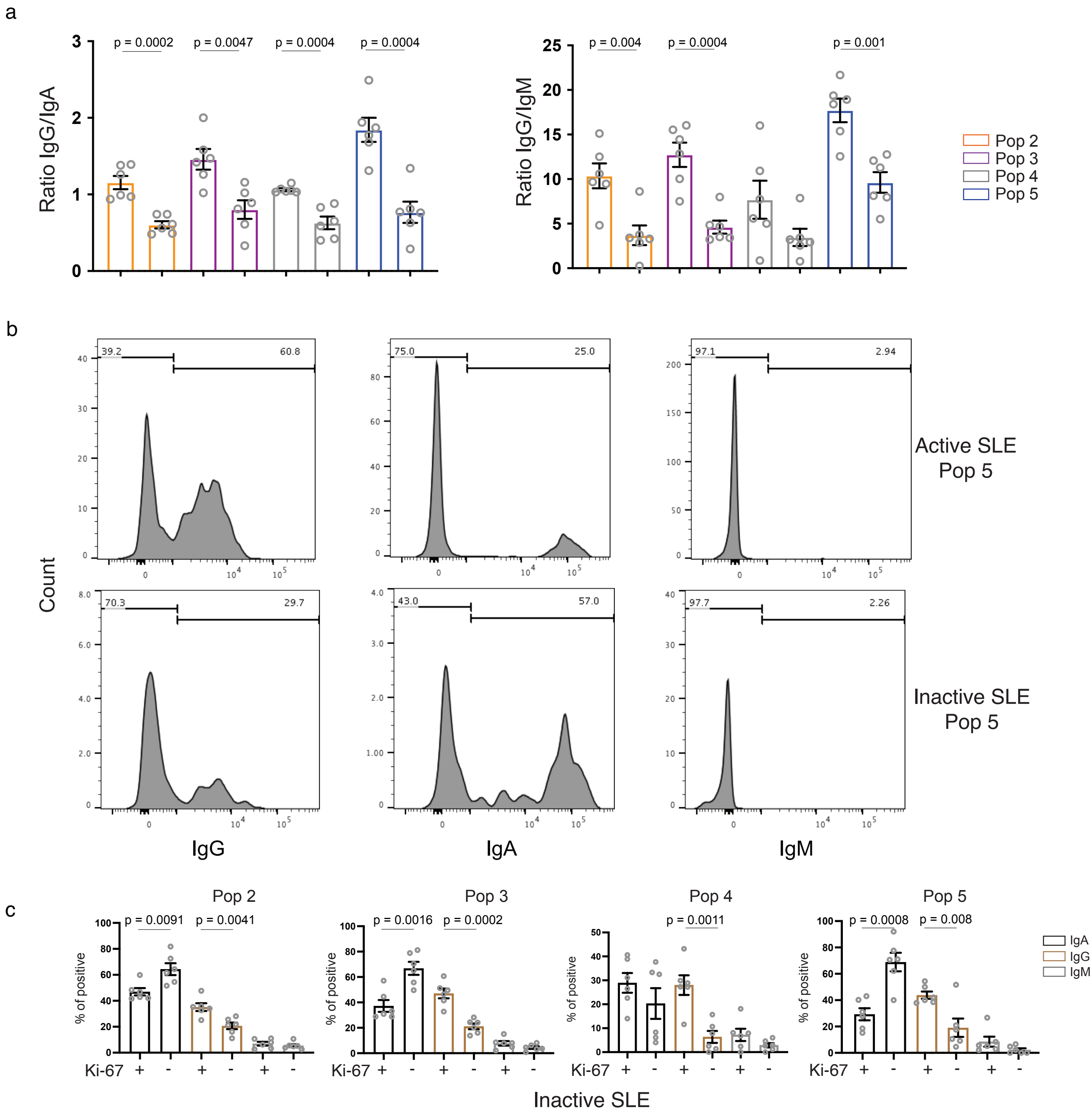

Supplementary Fig. 4: IgG-expressing ASC are enriched in active SLE patients. **a**, Intracellular staining of peripheral blood ASC from active SLE patients (*n* = 6) or inactive SLE patients (*n* = 6) with isotype-specific antibodies. Ratios of IgG/IgA and IgG/IgM in all ASC populations from either active or inactive SLE patients are shown as mean ± SEM. Statistical significance was assessed using Student's t test between disease groups within the same ASC population. **b**, Representative histograms show the expression of IgG, IgA and IgM in Pop 5 from both active and inactive SLE patients. **c**, The intracellular expression of IgA, IgG, and IgM in proliferating (Ki-67+) versus non-proliferating (Ki-67-) peripheral blood ASC from inactive SLE patients (*n* = 6). Data are shown as mean ± SEM. Statistical significance was assessed using Student's t test among disease groups within the same population. *p* values are shown on plots. Source data are provided as a Source Data file.

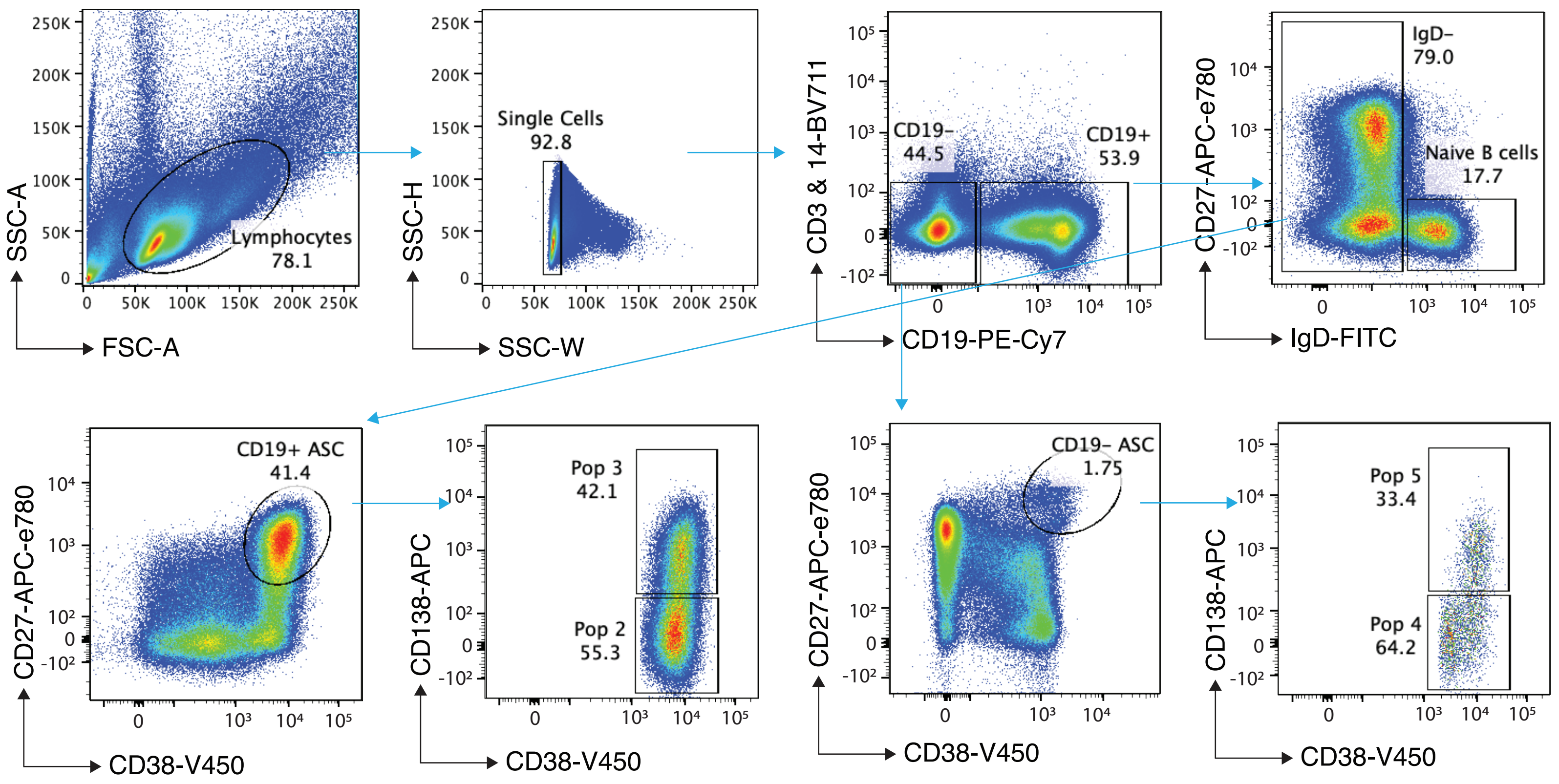

Supplementary Fig. 5: Gating strategy for ASC populations sorting (Pop 2, Pop 3, Pop 4, and Pop 5) for next gene sequencing, and for ASC populations as well as naive B cells for RNA sequencing.

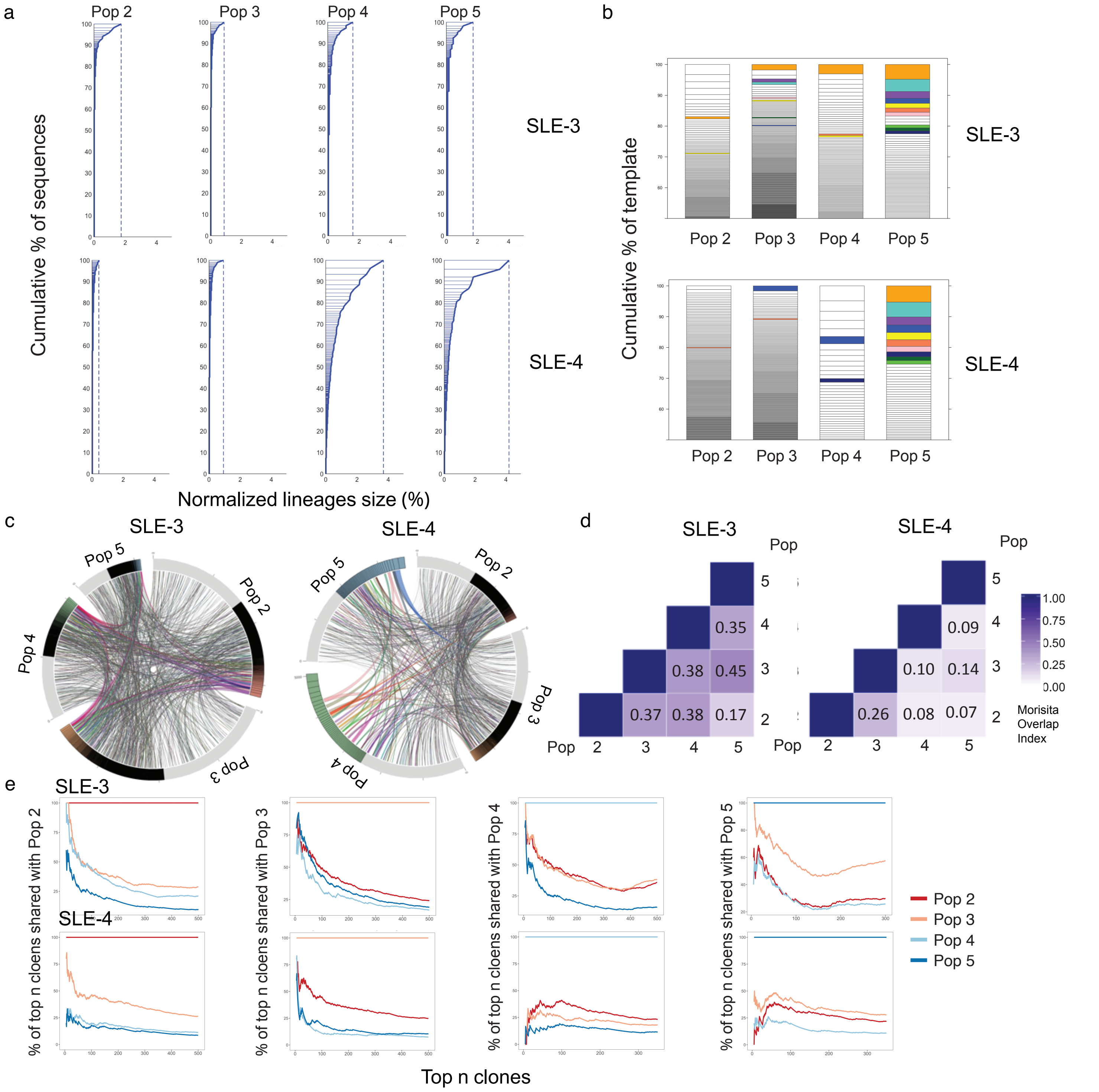

Supplementary Fig. 6: Heterogeneous SLE ASC responses share common precursors. AIRR-seq was used to analyze the clonal repertoire of ASC populations from 2 additional active SLE patients (see Fig. 5). **a**, Clonality of the repertoire in ASC populations is shown by plotting normalized lineage size versus the cumulative percent of sequences. Lineages are size-ranked in descending order along the extent of the y-axis representing 100% of all the sequences. Horizontal lines delineate the individual lineages. **b**, Stacked bar plots demonstrate the diversity of the repertoire by showing descending, size-ranked clones as segments comprising percentages of the total repertoire. The largest 10 clones of the reference population pop 5 are colored, and like-colors in other populations show identical clones in other populations. **c**, Circos plot shows interconnectedness of the ASC populations by plotting the sequences from each population in clonal size-ranked order with the largest clones being the most clockwise portion of each population segment. Lines between ASC populations indicate matched clones between ASC populations. **d**, The Morisita Overlap Index demonstrates the similarity of repertoires in various ASC populations as a value from 0 (no similarity) to 1 (identical repertoire). The color strength is indicative of interconnectivity. **e**, The clonal relatedness of SLE ASC populations is shown by plotting the percent of shared clones with Pop 2, Pop 3, Pop 4, and Pop 5, respectively (y axis), within the top numbers of clones (x axis).

a

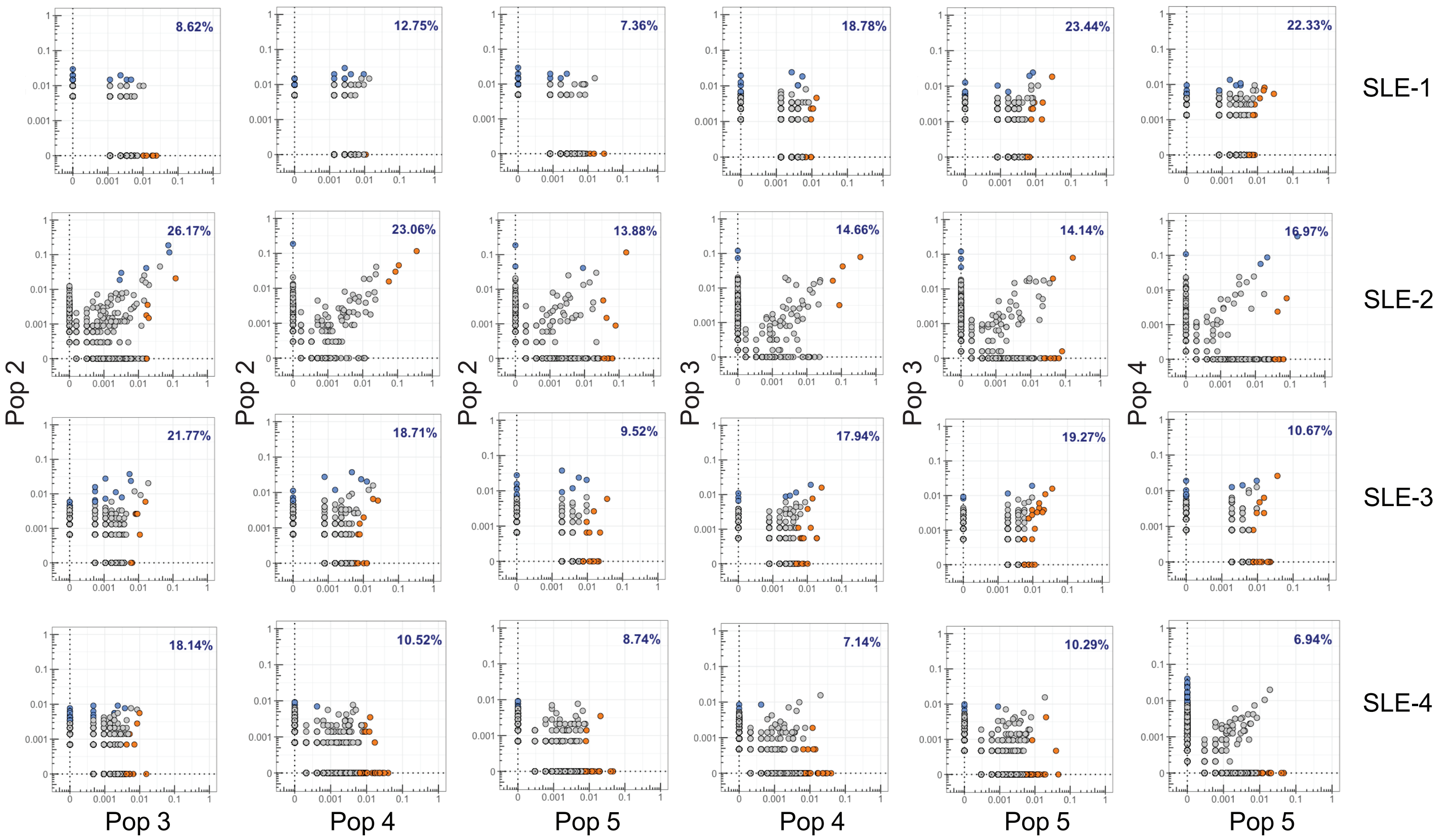

b

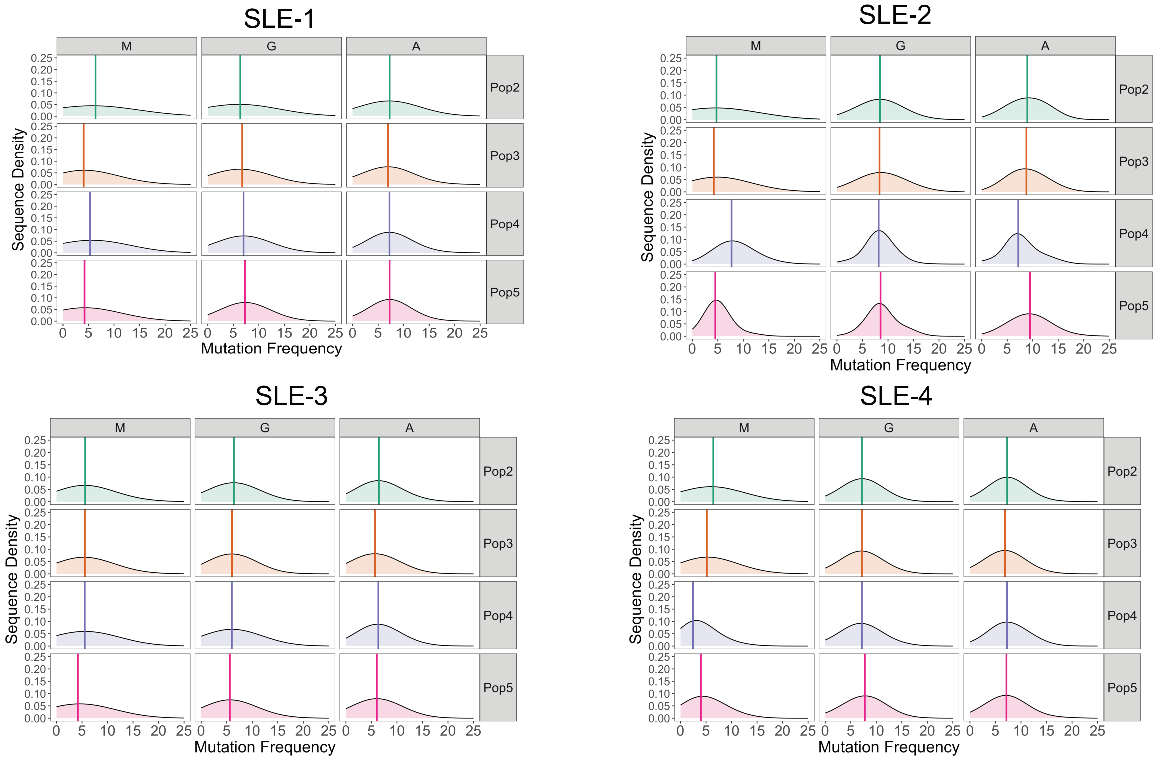

Supplementary Fig. 7: Clonal connectivity and somatic hypermutation analysis of SLE ASC. **a**, Scatter plots display clone frequencies of two populations that are shared (in the middle) or unique (on the axis). Clone frequencies that are significantly different between the populations are colored with orange (increased on the X axis population) or blue (increased on the Y axis population). Percent of clones that were found to be shared between populations is shown in the top right. **b**, Somatic hypermutation frequencies of each ASC population from four active SLE patients. The curve shows the density of sequences at each mutation frequency and the vertical line is the average for the ASC population expressing either IgM, IgG or IgA.

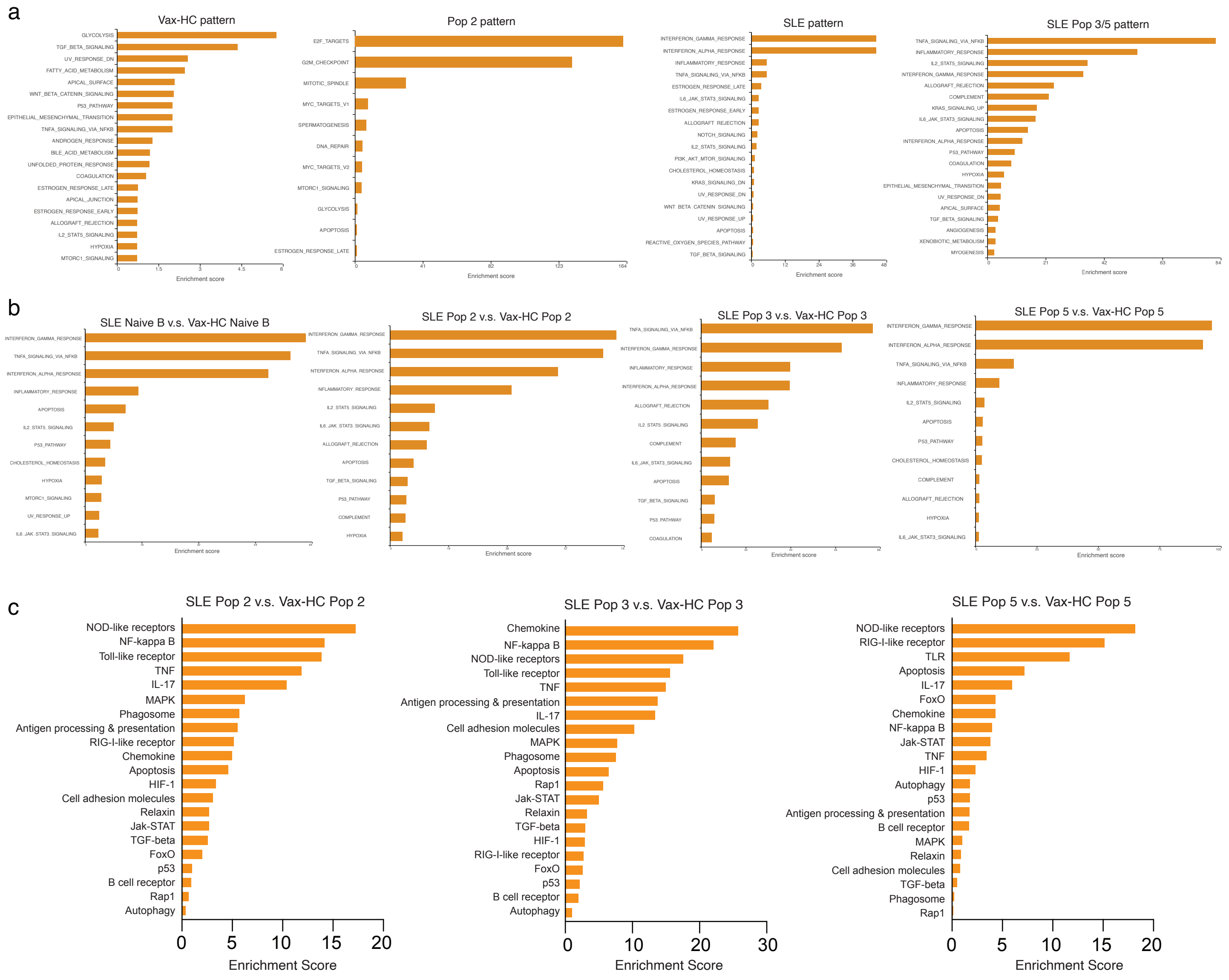

Supplementary Fig. 8: SLE ASC express abnormal pathways. **a**, GSEA pathways analysis shows the top pathways that are modulated in the four patterns identified in Fig. 6d. **b**, GSEA pathways analysis identifies the top pathways that are upregulated in ASC populations and naïve B cells from active SLE patients versus vaccinated healthy subjects. c, KEGG pathway analysis of DEG identified from the SLE and post-vax HC for each ASC populations.

		SLE-1	SLE-2	SLE-3	SLE-4
Cells	Pop 2	60,800	2,830	42,823	18,000
	Pop 3	18,850	1,040	34,783	10,000
	Pop 4	40,920	715	13,252	1,300
	Pop 5	22,000	435	18,574	1,100
Sequences	Pop 2	599	3,520	3,199	2,881
	Pop 3	1,758	6,413	3,837	3,523
	Pop 4	1,790	5,841	2,049	5,053
	Pop 5	2,090	4,982	1,097	3,635
Lineages	Pop 2	527	514	2,292	2,087
	Pop 3	1,305	563	2,816	2,194
	Pop 4	1,446	180	1,296	848
	Pop 5	1,395	231	863	839
D20	Pop 2	51	2	97	132
	Pop 3	79	3	155	106
	Pop 4	118	1	54	9
	Pop 5	60	2	66	11
D50	Pop 2	228	13	743	681
	Pop 3	427	18	941	530
	Pop 4	551	4	311	58
	Pop 5	350	9	341	82

Supplementary Table 1: Summary statistics of repertoire analysis for ASC from active SLE samples. ASC populations from four active SLE patients were used to analyze their clonal repertoire by using next generation sequencing. Numbers of sorted cells, of sequences and of lineages are listed for all populations in each individual. The D20 (D50) measure is the number of the largest lineages in a size-ordered list that span 20% (50%) of the sequences.
