# Supplementary material for: Distinct transcriptomes and autocrine cytokines underpin maturation and survival of antibody-secreting cells in systemic lupus erythematosus
Source: Nat Commun. 2024 Mar 1;15:1899. doi: 10.1038/s41467-024-46053-w (PMC10907730; doi:10.1038/s41467-024-46053-w)
Supplement: Supplementary file 3 — Reporting Summary [file 41467_2024_46053_MOESM3_ESM.pdf]

nature portfolio

Corresponding author(s): Ignacio Sanz, F. Eun-Hyung Lee

Last updated by author(s): Jan 12, 2024

Reporting Summary

Nature Portfolio wishes to improve the reproducibility of the work that we publish. This form provides structure for consistency and transparency in reporting. For further information on Nature Portfolio policies, see our Editorial Policies and the Editorial Policy Checklist.

Statistics

For all statistical analyses, confirm that the following items are present in the figure legend, table legend, main text, or Methods section.

n/a	Confirmed	
☐	☒	The exact sample size (*n*) for each experimental group/condition, given as a discrete number and unit of measurement
☐	☒	A statement on whether measurements were taken from distinct samples or whether the same sample was measured repeatedly
☐	☒	The statistical test(s) used AND whether they are one- or two-sided<br>*Only common tests should be described solely by name; describe more complex techniques in the Methods section.*
☐	☒	A description of all covariates tested
☐	☒	A description of any assumptions or corrections, such as tests of normality and adjustment for multiple comparisons
☐	☒	A full description of the statistical parameters including central tendency (e.g. means) or other basic estimates (e.g. regression coefficient) AND variation (e.g. standard deviation) or associated estimates of uncertainty (e.g. confidence intervals)
☐	☒	For null hypothesis testing, the test statistic (e.g. *F*, *t*, *r*) with confidence intervals, effect sizes, degrees of freedom and *P* value noted<br>*Give P values as exact values whenever suitable.*
☒	☐	For Bayesian analysis, information on the choice of priors and Markov chain Monte Carlo settings
☐	☒	For hierarchical and complex designs, identification of the appropriate level for tests and full reporting of outcomes
☐	☒	Estimates of effect sizes (e.g. Cohen's *d*, Pearson's *r*), indicating how they were calculated

Our web collection on statistics for biologists contains articles on many of the points above.

Software and code

Policy information about availability of computer code

Data collection	JEOL JEM-1400 TEM (JEOL Ltd) with Gatan US1000 2k x 2k CCD camera (Gatan), FlowJo 10.7.1, Image J 1.53
Data analysis	Prism 8.2.0, Partek Flow v10.0, Custom scripts and in-house developed analysis software were used with R, Matlab or Circos tools for visualization.

For manuscripts utilizing custom algorithms or software that are central to the research but not yet described in published literature, software must be made available to editors and reviewers. We strongly encourage code deposition in a community repository (e.g. GitHub). See the Nature Portfolio guidelines for submitting code & software for further information.

Data

Policy information about availability of data

All manuscripts must include a data availability statement. This statement should provide the following information, where applicable:

- Accession codes, unique identifiers, or web links for publicly available datasets
- A description of any restrictions on data availability
- For clinical datasets or third party data, please ensure that the statement adheres to our policy

The AIRR sequencing and RNA sequencing datasets for ASC populations were deposited at Genome Expression Omnibus under the accession number GSE235660. Source data are provided with this paper. Custom scripts and in-house developed analysis software were used with R, Matlab or Circos tools for visualization AIRR

sequencing data, and the scripts are available on GitHub at https://github.com/chenwr56/airr.

Research involving human participants, their data, or biological material

Policy information about studies with human participants or human data. See also policy information about sex, gender (identity/presentation), and sexual orientation and race, ethnicity and racism.

Reporting on sex and gender	Participants in this study are predominately female as SLE occurs much more frequent in women.
Reporting on race, ethnicity, or other socially relevant groupings	Our study population reflects the demographics of Atlanta, GA area and was predominately African American, a group that experiences higher SLE prevalence and severity. Both Black and white healthy control doors were used for comparison.
Population characteristics	Female, African American, age ranged from 22-65 years old.
Recruitment	SLE patients fulfilled four or more criteria of the modified American College of Rheumatology classification were recruited as active SLE patients, with less than four for SLE disease activity index were recruited as inactive SLE, both from Emory University Hospital and Grady Hospital. Healthy subject at steady state or post influenza vaccines were recruited based on matching gender, race and age with SLE patients.
Ethics oversight	All research was approved by the Emory University Institutional Review Board (Emory IRB numbers IRB00058515 and IRB00057983) and was performed in accordance with all relevant guidelines and regulations. Written informed consent was obtained from all participants.<br>Healthy donors (n = 45) were recruited using promotional materials approved by the Emory University Institutional Review Board. Healthy subjects received the influenza vaccinations (n = 24) as part of routine medical care. Subjects with systemic lupus erythematosus (n = 176) were recruited from Emory University Hospital and Grady Hospital in Atlanta, GA, USA. Peripheral blood mononuclear cells were isolated on days 6–7 after vaccination for all vaccinated subjects.

Note that full information on the approval of the study protocol must also be provided in the manuscript.

Field-specific reporting

Please select the one below that is the best fit for your research. If you are not sure, read the appropriate sections before making your selection.

☒ Life sciences ☐ Behavioural & social sciences ☐ Ecological, evolutionary & environmental sciences

For a reference copy of the document with all sections, see nature.com/documents/nr-reporting-summary-flat.pdf

Life sciences study design

All studies must disclose on these points even when the disclosure is negative.

Sample size	We collected as many samples as we can that met our recruitment criteria for each group in the period of time of this study.
Data exclusions	No data was excluded.
Replication	Flow cytometry experiments were conducted at least five human samples. Wright-Giemsa and electron microscopy were repeated for five times. ELISA were repeated for five times and each time with triplicate. ELISpot for cell survival experiments and neutralizing antibodies were conducted for five times and each time with triplicate. Attempts at replication were successful.
Randomization	Samples of participants were assigned to specific group based on their vaccination status and disease severity (see information above in the Recruitment).
Blinding	Blinding was performed wherever possible. Number systems, rather than sample IDs, were used for assays such as ELISA and ELISpot, and microscopy experiments, and data analysis for flow cytometry. All data were quantified independently to minimize bias.

Reporting for specific materials, systems and methods

We require information from authors about some types of materials, experimental systems and methods used in many studies. Here, indicate whether each material, system or method listed is relevant to your study. If you are not sure if a list item applies to your research, read the appropriate section before selecting a response.

Materials & experimental systems

n/a	Involved in the study
☐	☒ Antibodies
☒	☐ Eukaryotic cell lines
☒	☐ Palaeontology and archaeology
☒	☐ Animals and other organisms
☒	☐ Clinical data
☒	☐ Dual use research of concern
☒	☐ Plants

Methods

n/a	Involved in the study
☒	☐ ChIP-seq
☐	☒ Flow cytometry
☒	☐ MRI-based neuroimaging

Antibodies

Antibodies used

CD3-BV711 (clone: HIT3a, BD Biosciences, catalogue number: 740768, dilution at 1:20); CD14-BV711 (clone: M5E2, BD Biosciences, catalogue number: 740773, dilution at 1:20); IgD-FITC (IA6-2, BD Biosciences, catalogue number: 555778, dilution at 1:5); CD19-PE-Cy7(clone: SJ25C1, BD Biosciences, catalogue number: 341093, dilution at 1:5); CD27-APC-eFluor780 (clone: O323, Invitrogen, catalogue number: 50-161-60, dilution at 1:20); CD38-V450 (clone: HIT2, BD Biosciences, catalogue number: 561378, dilution at 1:20); CD138-APC (clone: 44F9, Miltenyi Biotec, catalogue number: 130-127-977, dilution at 1:20); CXCR4-PE (clone: 12G5, BioLegend, catalogue number: 306506, dilution at 1:20); CXCR3-PE (clone: G025H7, BioLegend, catalogue number: 352706, dilution at 1:20); Blimp-1-PE (clone: 6D3, BD Biosciences, catalogue number: 564702, dilution at 1:10); BCMA-PE (clone: 19F2, BioLegend, catalogue number: 357504, dilution at 1:10), IL-6R-PE (clone: M5, BD Biosciences, catalogue number: 561696, dilution at 1:20), BCL-2-PE (clone: Bcl-2/100, BD Biosciences, catalogue number: 340576, dilution at 1:10).

Validation

Antibodies were used for flow cytometry data analysis as well FACS sorting and data generated include Figure 1, 2, 3 (for analysis) and Figure 4, 5, 6 and 7 for sorting.

Flow Cytometry

Plots

Confirm that:

☒ The axis labels state the marker and fluorochrome used (e.g. CD4-FITC).

☒ The axis scales are clearly visible. Include numbers along axes only for bottom left plot of group (a 'group' is an analysis of identical markers).

☒ All plots are contour plots with outliers or pseudocolor plots.

☒ A numerical value for number of cells or percentage (with statistics) is provided.

Methodology

Sample preparation

Cells were isolated from peripheral blood using Ficoll density gradient centrifugation and perioheral blood mononuclear cells (PBMC) were stained with anti-human antibody staining reagents.

Instrument

FACSAria II (BD Biosciences)

Software

BD FACSDiva

Cell population abundance

1,000 to 5,000 were collected from sorting for each cell populations and post-sort purity analysis were performed to make sure the percentages of desired cells in specific sorting gate reach above 95%.

Gating strategy

To delineate the peripheral blood ASC, we divided CD3- and CD14-negative lymphocytes, i.e., non-T cells, non-monocytes, into CD19+ and CD19- subpopulations, and then CD19+ cells were gated on IgD-negative cells to eliminate late transitional and naïve B cells, followed by a subsequent gate on cells that express high levels of CD27 and CD38 for CD19+ ASCs. CD19+ ASCs were further defined by the expression of CD138 and grouped into pop 2 (CD19+ CD138-) and pop 3 (CD19+ CD138+). Likewise, CD19- ASCs were gated and further divided into pop 4 (CD19- CD138-) and pop 5 (CD19- CD138+) (Fig. 1b).

☒ Tick this box to confirm that a figure exemplifying the gating strategy is provided in the Supplementary Information.
